# Association between maternal haemoglobin status during pregnancy and children’s mental and psychomotor development at 18 months of age: Evidence from rural Bangladesh

**DOI:** 10.1080/16549716.2024.2390269

**Published:** 2024-08-28

**Authors:** Sayedur Rahman, Lina Wallberg, Anisur Rahman, Eva-Charlotte Ekström, Maria Kippler, Jena D Hamadani, Syed Moshfiqur Rahman

**Affiliations:** a Global Health and Migration Unit, Department of Women’s and Children’s Health, Uppsala University, Uppsala, Sweden; b Maternal and Child Health Division, International Centre for Diarrhoeal Disease Research, Dhaka, Bangladesh; c Institute of Environmental Medicine, Karolinska Institutet, Stockholm, Sweden

**Keywords:** Bayley test, haemoglobin, mental and psychomotor development, children, Bangladesh

## Abstract

**Background:**

Anaemia is commonly caused by iron deficiency and screened by haemoglobin (Hb) concentration in blood. There is a scarcity of longitudinal data on the relationship between maternal Hb levels during pregnancy and neurodevelopment in children.

**Objective:**

To measure the relationship of maternal Hb concentrations during pregnancy on early child development.

**Methods:**

This prospective cohort study included 1,720 mother-child dyads in rural Bangladesh. Maternal Hb concentrations were measured at 14 and 30 weeks of gestation. The child’s Mental Developmental Index (MDI) and Psychomotor Developmental Index (PDI) at 18 months of age were measured using Bayley Scales of Infant and Toddler Development (BSID-II). Data on socio-demographic characteristics, anthropometrics, mothers’ IQ and children’s home stimulation were also collected. Bivariate and multivariable-adjusted linear regression analyses were used to explore associations of maternal Hb with child development.

**Results:**

Mean Hb concentrations at 14 and 30 weeks of gestation were 116.6 g/L (±12.7) and 114.7 g/L (±12.7), respectively. Mean MDI and PDI scores among 18-month-old children were 78.9 (±12.4) and 93.8 (±13.7), respectively. Maternal 14-week Hb concentration was correlated with PDI (*r* = 0.06; *p* < 0.05) and 30-week Hb concentrations was correlated with MDI (*r* = 0.05; *p* < 0.05). Multivariable adjusted linear regression analysis showed that an increase in 14-week Hb concentrations increased the PDI scores among boys (β = 0.09; 95% CI: 0.02, 0.16). Hb concentrations at 30 weeks of gestation were not associated with MDI or PDI scores.

**Conclusion:**

Higher maternal Hb concentrations at 14 weeks of gestation were associated with higher PDI among 18-month-old boys in Bangladesh.

## PAPER CONTEXT

- **Main findings:** Observational studies reported that low haemoglobin concentration during pregnancy is associated with poor neurodevelopmental outcomes among offspring; however, population-based longitudinal data are scarce.
- **Added knowledge:** Our study conducted in rural Bangladesh suggests that higher maternal haemoglobin levels in early pregnancy (14 weeks of gestation) are positively associated with psychomotor performance in boys.
- **Global health impact for policy and action:** The study finding provides evidence to inform public health policies aimed at improving maternal and child health outcomes, particularly in regions with high rates of anaemia during pregnancy.

## Background

Nutritional status during pregnancy is a fundamental determinant of maternal health, pregnancy outcomes, and foetal growth and development [1,2]. Maternal micronutrient deficiency, particularly iron deficiency during pregnancy, is widespread and recognised as a major public health concern in low-and-middle-income countries [3]. Iron deficiency is the most common nutritional cause of anaemia, although deficiencies in folic acid, vitamin A and B12, and infectious diseases including malaria, HIV/AIDS, helminthiasis, and tuberculosis are also important causes [4]. To screen for anaemia as a proxy for iron deficiency during pregnancy, maternal haemoglobin (Hb) concentration in blood is the most commonly used biomarker, particularly in resource-poor settings, because of its low cost, the ease and speed of the procedure, and performance [5]. The World Health Organization (WHO) defines anaemia in pregnant women as a Hb concentration <110 g/L and has included maternal anaemia in the core set of indicators for the Global Nutrition Monitoring Framework [4].

There is accumulating evidence suggesting that a low Hb concentration during pregnancy is associated with adverse birth outcomes such as stillbirth, preterm birth, low birth weight, and small-for-gestational-age [6–8]. More recent studies even reported that both low and high Hb concentrations during pregnancy could serve as significant risk factors for adverse birth outcomes [9,10]. Many studies have also investigated the impact of iron status on cognitive, motor, and social-emotional development in toddlers and young children in many settings as iron is essential for proper neurogenesis, neurometabolism, neurotransmission, and myelination [11,12]. However, a vast majority of these studies evaluated the neurodevelopmental outcomes among iron-deficient anaemic infants [13–15]. Fewer studies have assessed the longitudinal effects of maternal gestational iron status on cognitive and motor development during early childhood, and findings have been equivocal [14,16–18]. Furthermore, most of the previous studies that evaluated the impact of maternal gestational iron status focused on the effect of anaemia, defined either by its presence or absence or by other categorical definitions, and did not evaluate the child neurodevelopmental risks associated with high or low maternal Hb concentrations during pregnancy.

The aim of this study was to assess the impact of maternal Hb levels measured on a continuous scale at two different time points during pregnancy on mental and psychomotor development of their children at 18 months of age in a rural Bangladeshi cohort where the prevalence of anaemia is high and early childhood development is neglected.

## Methods

### Study area and population

The study was conducted in Matlab, a rural sub-district situated 57 km south of the capital city Dhaka, Bangladesh. In this area, the International Centre for Diarrhoeal Disease Research, Bangladesh (icddr,b), has been operating a Health and Demographic Surveillance System since 1966. The study was nested into a large population-based cohort study titled ‘Maternal and Infant Nutrition Intervention in Matlab’ (MINIMat Trial; Reg# ISRCTN16581394) for which the design and procedures can be found elsewhere [19]. The MINIMat trial enrolled 4,436 pregnant women in the study areas between November 2001 and December 2003. A subsample (*n* = 2,853) of MINIMat trial participants comprising all pregnant women who gave birth to a live-born singleton infant between May 2002 and December 2003 were selected for developmental assessments of their offspring at the age of 18 months.

## Measurements

### Maternal Hb concentrations during pregnancy

All participating pregnant women were scheduled to visit nearby health centres for antenatal check-ups by nurses. At the health centres, women’s Hb concentrations in venous blood were measured (unit of measurement: g/L) at average gestational weeks (GW) 14 (total range 8–24; 5^th^-95^th^ percentiles = 11–17) and 30 (total range 23–40; 5^th^-95^th^ percentiles = 27–34) by using HemoCue Photometer (HemoCue AB, Ängelholm, Sweden). Measuring Hb concentration at 14 weeks provides a baseline assessment early in pregnancy before significant hemodilution occurs, ensuring identification of pre-existing anaemia. By week 30, the plasma volume expansion has usually stabilised, and Hb levels reflect the maternal adaptation to pregnancy and can identify anaemia that may impact foetal growth and third-trimester outcomes [20].

### Child developmental assessments at 18 months

Child’s Mental Developmental Index (MDI) and Psychomotor Developmental Index (PDI) were measured using the revised version of the Bayley Scales of Infant and Toddler Development (BSID-II) [21]. Globally, Bayley test is widely used to assess a child’s cognitive, language, and motor development, and it has previously been used in many research studies conducted in Bangladesh [22–27]. In the MINIMat study, five psychologists/testers were trained to conduct the assessments at local healthcare facilities when the children turned 18 months of age. The child’s MDI and PDI were measured at 18 months of age. At this age, children typically show significant developmental milestones, making it an ideal time to evaluate cognitive and motor skills that can provide valuable insights into their overall growth and development [28]. Prior to the commencement of the assessments, the inter-observer reliability of the five testers with the trainer was conducted with ten children each, and the intraclass correlations ranged from *r* = 0.88 to 0.99 for both MDI and PDI.

### Covariates

Maternal body mass index (BMI, kg/m^2^) was calculated based on women’s weight and height measured at enrolment (i.e. at around 9 GW). Child body weight was measured at birth and at 18 months using an electronic balance beam that was precise to ±10 gm. Child height was measured to the nearest 0.1 cm with locally produced length boards. The head circumference was measured with non-stretchable tape to the nearest 1 mm [25]. All the anthropometric measurements were taken by trained community health research workers according to the standard operating procedures and guidelines developed by the WHO. Child height and weight were converted to standard z-scores using the WHO Child Growth Standards [29].

Socio-economic status (SES) information including the age of the mother, parental education and occupation, and structure of the house were collected through home interviews by the community health research workers. Wealth index as a measure of SES was created from the information on the possession of certain household items (e.g. television, radio, domestic animal, chair, table, bed, bicycle, rickshaw, etc.) using principal component analysis [30]. Households were divided into SES tertiles based on the wealth index (poorest, middle-class, and wealthiest). Maternal educational attainment was categorised according to the number of formal years of education: no education, 1–5 years, ≥6 years. Parity was determined based on the number of live-born children before the index pregnancy and was dichotomised into primiparity (no child) and multiparity (≥1 child). Gestational age at birth (in weeks) was calculated by subtracting the first date of the last menstrual period (LMP) from the date of delivery and was dichotomised into preterm (<37 weeks) and term (≥37 weeks) deliveries.

Home Observations for Measurement of the Environment (HOME) were also conducted to assess the dimensions of the home environment including the quality of stimulation and learning opportunities.

### Statistical analysis

All the statistical analyses were performed using Stata 13 SE (StataCorp. 2013. Stata Statistical Software: Release 13. College Station, TX: StataCorp LP). The population characteristics were presented as either mean and standard deviation (SD) or frequencies for numerical and categorical data, respectively. Student’s t-test was used for comparing the means and Pearson’s Chi-squared test was used to assess the relationship between two categorical variables. An association was considered statistically significant if the *p*-value was <0.05. As data were not normally distributed, bivariate analyses were performed using Spearman’s Rank Correlation Test and expressed as correlation coefficient *(r)*. To evaluate associations of maternal Hb status at 14 and 30 weeks during pregnancy with child MDI and PDI at 18 months of age, multivariable linear regression analyses adjusted for potential covariates were performed. The inclusion of confounding factors in the multivariate model was guided by findings from bivariate analysis (*p*-value <0.05) as well as knowledge from the existing literature. Associations were expressed as β-coefficient and corresponding 95% confidence intervals (CIs). All tests were two-sided, and an association was considered significant if the 95% CI did not include zero.

## Results

**Figure 1.**
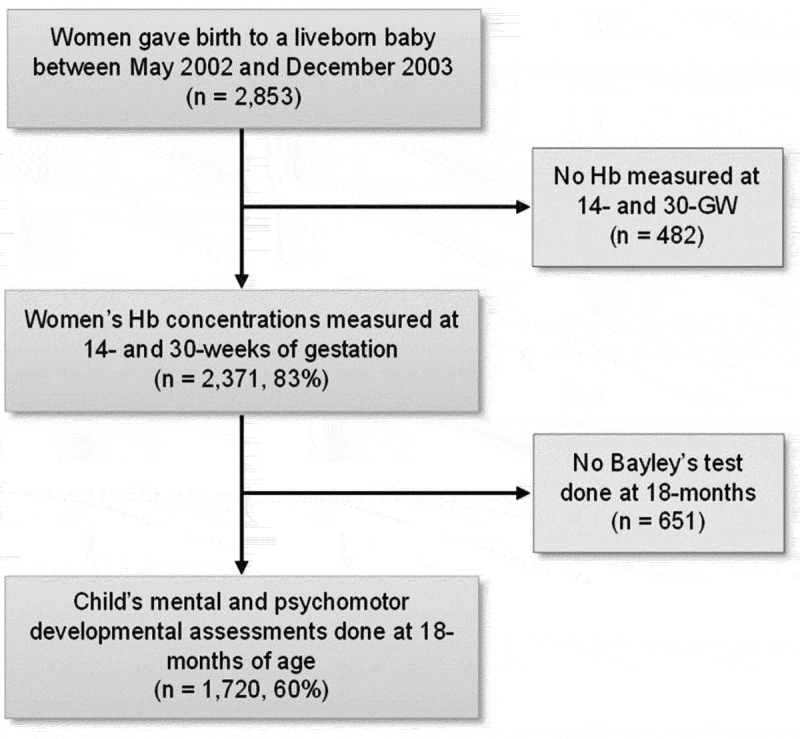
Study sample selection flow diagram.

Of the total 2,853 mother-child dyads, Hb concentrations in blood were measured at 14 weeks and 30 weeks of pregnancy for 2,371 (83%) mothers. Out of these 2,371 children, 1,720 (60% of the total participants) had their developmental assessments including MDI and PDI done at 18 months of age (Figure 1) and were included in the present analysis.

Background characteristics of the 1,720 mother-child pairs are described in Table 1. The mean age of the mothers was 26.4 years (SD ± 5.9). One-third of the mothers were illiterate, and 487 (28.3%) had a BMI <18.5 kg/m^2^. Around 30% of the mothers (*n* = 518) were anaemic (Hb <110 g/L) at 14 weeks of gestation. The mean ± SD Hb concentration at 14 weeks significantly differed between mothers who delivered a boy (117.2 g/L ± 12.3) and those who delivered a girl (115.9 g/L ± 13.1; *p*-value 0.031). A higher proportion (34.2%) of anaemic mothers were observed at 30 weeks of pregnancy, which is indicative of a decrease in the Hb concentrations in maternal blood as the pregnancy progresses. However, there was no difference in mean maternal Hb concentrations at 30 weeks of pregnancy by child sex.

**Table 1.** Background characteristics of mothers and children.

Characteristics*	All	Child sex		*p*-value^† ¥^
		Boys	Girls	
**Number of participants**	1720	895	825	
**Maternal characteristics**				
Age (years)	26.4 (**±**5.9)	26.3 (**±**5.8)	26.4 (**±**6)	0.863
BMI (kg/m^2^)	20.2 (**±**2.7)	20.2 (**±**2.7)	20.1 (**±**2.7)	0.865
Education (years)				0.812
No education	561 (32.6)	292 (32.6)	269 (32.6)	
1 to 5	377 (21.9)	191 (21.3)	186 (22.5)	
≥6	782 (45.5)	412 (46)	370 (44.8)	
Parity				0.311
Primiparous	571 (33.2)	307 (34.3)	264 (32)	
Multiparous	1149 (66.8)	588 (65.7)	561 (68)	
Socioeconomic status (SES)^£^				0.769
Poorest	585 (34)	298 (33.3)	287 (34.8)	
Middle	545 (31.7)	284 (31.7)	261 (31.6)	
Wealthiest	590 (34.3)	313 (35)	277 (33.6)	
Hb level (g/L) at GW14	116.6 (**±**12.7)	117.2 (**±**12.3)	115.9 (**±**13.1)	**0.031**
Hb level (g/L) at GW30	114.7 (**±**12.7)	115.1 (**±**12.4)	114.3 (**±**13)	0.195
Mother’s IQ (total Ravens score)	25.2 (**±**12)	25.4 (**±**12.1)	24.9 (**±**11.8)	0.363
**Birth measures**				
Gestational age (weeks)	38.7 (**±**1.6)	38.6 (**±**1.7)	38.9 (**±**1.5)	**<0.001**
Head circumference (cm)	32.5 (**±**1.6)	32.7 (**±**1.7)	32.2 (**±**1.5)	**<0.001**
WAZ	−1.35 (±0.93)	−1.34 (±0.94)	−1.36 (±0.91)	0.791
HAZ	−0.9 (**±**1.09)	−0.95 (**±**1.13)	−0.86 (**±**1.05)	0.089
WHZ	−1.02 (**±**1.06)	−0.99 (**±**1.06)	−1.05 (**±**1.07)	0.337
**18-month measures**				
Age (months)	18.0 (**±**0.14)	18.0 (**±**0.14)	18.0 (**±**0.14)	0.441
WAZ	−1.6 (**±**1)	−1.6 (**±**1)	−1.5 (**±**1)	0.388
HAZ	−1.9 (**±**1.1)	−2 (**±**1.1)	−1.9 (**±**1)	0.078
WHZ	−0.8 (**±**1)	−0.9 (**±**1)	−0.8 (**±**1)	0.407
MDI	78.9 (**±**12.4)	78.9 (**±**12)	78.8 (**±**12.8)	0.946
PDI	93.8 (**±**13.7)	93.5 (**±**13.5)	94.1 (**±**13.8)	0.358
Total HOME	83.7 (**±**7.1)	84.1 (**±**7.2)	83.2 (**±**7)	**0.009**

Abbreviations: GW, gestational age in weeks; Hb, haemoglobin; SES, socioeconomic status; BMI, body mass index; WAZ, weight for age z-score; HAZ, height/length for age z-score; WHZ, weight for height/length z-score; HOME, home observation for measurement of environment.

*Data are number (column percentage) or mean (**±**SD).

^**†**^*p*-value calculated by performing Student’s t-test or Pearson’s Chi-squared test.

^£^SES: based on a number of wealth indices (range= −5 to + 5).

^¥^Significant at *p* <0.0

The average gestational age at birth was 38.7 weeks (range 30–44 weeks), and 11% of the newborns were born preterm (<37 weeks). The mean ± SD birth weight was 2,707 ± 389 gm (range 1250–4150 gm) for all children, and boys were significantly heavier than girls (*p*-value <0.001). Also, at 18 months of age, boys were heavier (9.2 ± 1.1 kg vs. 8.6 ± 1.1 kg; *p*-value <0.001) and taller (76.8 ± 3.0 cm vs. 75.1 ± 2.9 cm; *p*-value <0.001) than the girls. There were no differences in mean MDI and PDI scores at 18 months of age between boys and girls. The HOME scores, on the other hand, varied significantly, with a mean ± SD score of 84.1 ± 7.2 in boys versus 83.2 ± 7.0 in girls (*p*-value 0.008).

Bivariate analysis showed that maternal Hb concentration at 14 weeks of gestation was positively correlated with child’s PDI scores (*r* = 0.06, *p* = 0.01) (Table 2). On the other hand, Hb concentration at 30 weeks of gestation was found to be positively correlated with child’s MDI scores (*r* = 0.05, *p* = 0.04). Maternal IQ was significantly positively correlated with both child’s MDI (*r* = 0.18, *p* < 0.001) and PDI scores (*r* = 0.09, *p* < 0.001). Maternal education (*r* = 0.21, *p* < 0.001) and SES (*r* = 0.26, *p* < 0.001) were positively correlated with child’s MDI scores, whereas parity (*r* −0.2, *p* < 0.001) was negatively correlated with MDI. Similar correlations with child’s PDI scores were observed for maternal education (*r* = 0.15, *p* < 0.001), SES (*r* = 0.17, *p* < 0.001), and parity (*r* =-0.07, *p* < 0.001). All the child anthropometric measurements at birth and at 18 months of age were correlated with MDI and PDI scores except for WHZ at birth which was not significantly correlated with PDI (Table 2).

**Table 2.** Bivariate associations of maternal characteristics during pregnancy and child characteristics at birth and 18 months of age with child’s mental and psychomotor developmental outcomes at 18 months of age (*n* = 1,720).

Variables	Child developmental assessments^§^	
	MDI	PDI
**Maternal characteristics**		
Age	−0.11**	−0.03
Education	0.21**	0.15**
Parity	−0.2**	−0.07**
SES	0.26**	0.17**
BMI	0.09**	0.1**
Hb level (g/L) at GW14	0.02	0.06*
Hb level (g/L) at GW30	0.05*	0.01
Mother’s IQ	0.18**	0.09**
**Child anthropometry at birth**		
WAZ	0.14**	0.12**
HAZ	0.1**	0.11**
WHZ	0.08**	0.04
Head circumference	0.09**	0.12**
Gestational age	0.08**	0.11**
**Child anthropometry at 18 months**		
Age	0.04	0.05*
WAZ	0.24**	0.23**
HAZ	0.24**	0.26**
WHZ	0.17**	0.14**
Total HOME	0.26**	0.18**

SES, socioeconomic status; BMI, body mass index; WAZ, weight for age z-score; HAZ, height/length for age z-score; WHZ, weight for height/length z-score; HOME, home observation for measurement of environment.

Data are correlation coefficients *(r)*.

^**§**^*r* was calculated by performing Spearman’s Rank Correlation Test.

*Significant at *p* <0.05.

**Significant at *p* <0.01.

In the crude linear regression model, an association between maternal Hb concentration at 14-GW and MDI score was observed in boys (β = 0.06; 95% CI: 0, 0.13; *p*-value = 0.047), but not in girls (β = −0.00; 95% CI: −0.07, 0.07; *p*-value = 0.991) (Table 3). Maternal Hb concentrations at 30-GW were found to be associated with MDI scores among all children in the crude model (β = 0.05; 95% CI: 0.01, 0.09; *p*-value = 0.026). Nonetheless, after adjusting for selected confounders, neither maternal Hb concentrations at 14-GW nor 30-GW were associated with MDI scores at 18 months of age in all children or in the sex stratified models (Table 3).

**Table 3.** Linear regression analyses of maternal Hb concentrations at 14 and 30 weeks during pregnancy with the child’s mental development (MDI) at 18 months.

	Crude model			Adjusted model*		
	β	95% CI	*p*-value^¥^	β	95% CI	*p*-value^¥^
**14-GW Hb**						
All children	0.03	(−0.01, 0.08)	0.181	0.01	(−0.04, 0.05)	0.767
Boys	0.06	(0, 0.13)	**0.047**	0.05	(−0.01, 0.11)	0.097
Girls	−0.00	(−0.07, 0.07)	0.991	−0.03	(−0.1, 0.03)	0.281
**30-GW Hb**						
All children	0.05	(0.01, 0.09)	**0.026**	0.02	(−0.02, 0.07)	0.335
Boys	0.05	(−0.01, 0.11)	0.124	0.02	(−0.04, 0.08)	0.501
Girls	0.05	(−0.01, 0.12)	0.108	0.03	(−0.04, 0.09)	0.425

β, regression coefficient; CI, confidence interval; GW, gestational week.

*Adjusted for maternal age, maternal education, HOME, and child sex (excluded in the stratified model) and height-for-age z score at 18 months.

^¥^Significant at *p* <0.05

Maternal Hb concentrations at 14-GW were positively associated with the children’s PDI scores at 18 months in the crude model (β = 0.06; 95% CI: 0.01, 0.11; *p*-value = 0.020), and after stratification by child sex, this association was only evident in boys (β = 0.11; 95% CI: 0.03, 0.18; *p*-value = 0.004) (Table 4). After adjusting the models for confounders, the association was no longer significant for all children. However, the estimate of Hb concentrations at 14-GW with PDI score was still significant in boys (β = 0.09; 95% CI: 0.02, 0.16; *p*-value = 0.009), however not in girls. No association was observed between maternal Hb concentration at 30-GW with children’s PDI scores at 18 months of age.

**Table 4.** Linear regression analyses of maternal hb concentrations at 14 and 30 weeks during pregnancy with child’s psychomotor development (PDI) at 18 months.

	Crude model			Adjusted model*		
	β	95% CI	*p*-value^¥^	β	95% CI	*p*-value^¥^
**14-GW Hb**						
All children	0.06	(0.01, 0.11)	**0.020**	0.04	(−0.01, 0.09)	0.075
Boys	0.11	(0.03, 0.18)	**0.004**	0.09	(0.02, 0.16)	**0.009**
Girls	0.02	(−0.05, 0.09)	0.609	0.00	(−0.07, 0.07)	0.968
**30-GW Hb**						
All children	0.01	(−0.04, 0.06)	0.590	0.01	(−0.04, 0.06)	0.820
Boys	0.05	(−0.02, 0.12)	0.147	0.03	(−0.04, 0.11)	0.336
Girls	−0.02	(−0.09, 0.05)	0.535	−0.02	(−0.09, 0.05)	0.603

β, regression coefficient; CI, confidence interval; GW, gestational week.

*Adjusted for maternal age, maternal education, HOME, and child sex (excluded in the stratified model) and height-for-age z score at 18 months.

^¥^Significant at *p* <0.05

## Discussion

In this prospective, population-based cohort study, maternal Hb concentrations at 14 weeks of gestation were found to be positively associated with boys’ psychomotor performance. A similar association, although statistically non-significant, was observed in relation to the boy’s mental development at 18 months of age. We found no evidence that maternal Hb concentrations at 30 weeks of gestation influenced a child’s MDI and PDI scores in the study cohort. To the best of our knowledge, this is the first longitudinal study from Bangladesh that evaluated these associations using prenatal Hb levels in a continuous manner.

Overall, the positive associations observed in this study, even though mostly nonsignificant, suggest that higher maternal Hb concentrations during pregnancy may lead to improved mental and psychomotor development among children of 18 months of age. Our findings are consistent with several previous studies that have examined the effects of maternal Hb concentrations, iron deficiency, or iron deficiency anaemia (IDA) during pregnancy on the mental and psychomotor development of young children. From a large longitudinal, prospective birth cohort study conducted in Uganda, Nampijja et al. [14] reported that lower maternal Hb levels during pregnancy were associated with reduced psychomotor (fine motor + gross motor) scores among young children at 15 months of age (β = 0.05; 95% CI: 0.0002, 0.09; *p*-value = 0.05). Similar associations were observed in a prospective cohort study carried out in rural Vietnam [31]. The authors of this study reported that maternal anaemia (Hb <11 g/dL) at around 28 weeks of gestation had direct adverse effects on BSID motor development scores in Vietnamese infants (mean reduction of 2.61 points; 95% CI: 0.57, 4.65). Another recent study conducted in Vietnam [32] reported that offspring born to women with low initial Hb-decline during pregnancy had lower motor development scores at 12 months of age. However, this association was no longer significant after adjusting for multiple comparisons and was not observed at 24 months of age. Maternal Hb trajectories were also not associated with child cognition or language at 12 or 24 months of age in adjusted models. A cohort study conducted in Benin, however, reported an inverted U-shaped relationship between maternal Hb levels and infant gross motor scores. The researchers observed that infant gross motor scores increased sharply with increasing maternal Hb concentration until 90 g/L and then began to fall after 110 g/L. They concluded that a maternal Hb concentration range of 90–110 g/L might be optimal for improved motor function among the infants [17]. In a study of 278 children of 5-year age in Birmingham, Alabama, low cord serum ferritin concentrations were found to be associated with poorer scores in full-scale intelligence quotient, language ability, fine- and gross-motor skills, attention, and tractability [33]. A double-blind cluster randomized controlled trial of prenatal folic acid, iron/folic acid, and multiple micronutrient supplementation among 850 women in western China, reported a significantly lower MDI among their offspring at 12, 18, and 24 months of age in the prenatal-IDA group compared to the non-IDA group of women [34]. The study, however, did not find an impact of prenatal iron status on PDI in young children. Noteworthily, while many studies reported negative effects of lower maternal Hb concentrations or iron deficiency during pregnancy on a child’s neurodevelopment in the mental and motor domains, several other epidemiological studies did not find an association between them [16,35].

Several experimental studies in animal models provided evidence of impairments in learning, memory function and behavioral changes among offspring exposed to inadequate prenatal iron levels [36–39]. In humans, brain growth spurt begins in the third trimester of pregnancy and continues to approximately 2 years after birth. Deficits accrued during this period are believed to have detrimental effects on the neuro-development of the foetus [11,12]. Iron deficiency during the early postnatal period has also been shown to have a lasting impact on gene regulation throughout one’s lifespan, resulting in cognitive impairment and neuropsychiatric disorders [40]. Nonetheless, solid mechanistic evidence on the role of iron status on offspring brain development and neurodevelopmental outcomes is sparse [11,41]. Potential factors that may play a pivotal role include dysfunctional myelination, neurotransmission disturbance, and endocrine pathways [42].

The complex pathophysiological mechanisms underlying sex-related differences in early childhood development are not well-understood, however, hypothetically can be explained by alteration of placental genes due to maternal iron deficiency. A recent animal model study reported gender-specific differences in placental responses to maternal iron deficiency, suggesting a possible dimorphic effect on the developing foetus [43]. In this study, placental tissue was isolated at E14 and transcriptomic and proteomic analyses of placental homogenates were undertaken and compared as a function of placental sex. Analysis of the RNA-Seq data identified six genes that were similarly up- (Tfrc, Slc11a2, Gypa, Hemgn) or down- (Tmcc2, Cts6) regulated in both the male and female placentas. However, an additional 154 differentially expressed genes were uniquely impacted by iron deficiency only in the male foetuses [43,44]. Poor foetal iron status (cord serum ferritin concentrations) is also reported to be associated with diminished performance in certain mental and psychomotor tests [33]. It is possible that maternal early-life iron/ferritin status exerts an influence on the sex differences in early childhood development. Findings from a recent study conducted by Guo et al. among 4,579 infants aged 6–12 months in Guangzhou, China, revealed a linear relationship between concurrent serum ferritin levels and general quotient, gross motor, fine motor, language, and adaptive behaviour scores in females, with no such relationship observed in males [45]. Furthermore, Loke et al. have reviewed studies of biological factors underlying sex dimorphism in several neurological disorders such as attention-deficit hyperactivity disorder (ADHD), schizophrenia, and autism spectrum disorders. The authors reported several sex-specific hormones and sex-specific genes as the underlying factors for sex differences in neurological disorders, implying that the impact can differ at different ages, affecting either boys or girls [46]. However, the critical window of exposure was not clear and the impact can differ by age. In the present study, sex differences were observed between maternal prenatal Hb levels and psychomotor development among offspring. However, as we neither measured the gene and protein signatures of iron-deficient maternal placentas nor the cord blood or infants’ iron/ferritin status, limited conclusion can be drawn regarding the above hypotheses on the causal pathways of sex-related differences in MDI as well as PDI scores that warrant further research.

This was a large, population-based study, including 1,720 children with maternal Hb concentrations measured at two time points during pregnancy as well as individual measures of child’s neurodevelopment. Furthermore, a comprehensive number of sociodemographic variables were assessed. The BSID-II has previously been adapted [47] and used in several studies and has demonstrated validity in this population [22–27,48]. In the present study, the BSID-II scores had good test–retest reliability and concurrent validity and were significantly correlated with height-for-age and the SES factors in theoretically expected ways. Although there were five psychologists who conducted the child development tests, they were blinded about the mother’s Hb status and were rotated around the study area throughout the study period as part of our efforts to minimise biases. A limitation of the study is that approximately 40% of participants from the original MINIMat cohort were excluded due to the unavailability of prenatal Hb data as well as children’s developmental scores at 18 months. However, when comparing potential differences in background characteristics of included and excluded women and children, the observed differences were very small (data not shown). Another limitation was the inability to investigate the effects of children’s Hb levels or anaemia as these parameters were not assessed in the children. Moreover, it is important to take into consideration that decreased maternal Hb levels during pregnancy can result from a variety of factors beyond iron deficiency, including infections, helminthiasis, haemoglobinopathies, and other nutritional deficiencies. Unfortunately, we lacked maternal data on plasma ferritin for a considerable portion of the mothers (*N* = 2,234), which is a more specific indicator of iron status than Hb, although it may also be sensitive to concurrent infection. Finally, it cannot be excluded that our studied associations could have been influenced by unmeasured residual confounding (e.g. household crowding or birth order of the index child).

## Conclusions

No clear association was observed between prenatal Hb concentrations and child’s mental and psychomotor development at 18 months of age. However, there was an exception for the 14 weeks’ Hb concentration and the PDI score among boys. We found no evidence that Hb concentrations during late pregnancy had an impact on child’s mental and psychomotor functions. Additional research is warranted to determine whether effects of maternal Hb concentrations during pregnancy on children’s development become apparent in similar settings.
